# Factors associated with impaired hypoglycemia awareness in patients with type 2 diabetes in Korea: a cross-sectional study

**DOI:** 10.7586/jkbns.25.054

**Published:** 2025-11-18

**Authors:** Ji Young Kim, Youngran Yang

**Affiliations:** 1 Department of Nursing, Wonkwang Health Science University, Iksan, Korea; 2 College of Nursing, Research Institute of Nursing Science, Jeonbuk National University, Jeonju, Korea

**Keywords:** Diabetes mellitus, type 2, Awareness, Hypoglycemia, Insulin

## Abstract

**Purpose:**

Impaired awareness of hypoglycemia (IAH) is characterized by a diminished ability to recognize the early symptoms of hypoglycemia, which in turn increases the risk of severe hypoglycemic episodes and related complications. This study aimed to identify factors associated with IAH among patients with type 2 diabetes mellitus (T2DM).

**Methods:**

A descriptive survey was conducted among 185 outpatients aged ≥ 19 years who had been diagnosed with T2DM and had received oral hypoglycemic agents or insulin therapy for more than 6 months. IAH was assessed using the Clarke Questionnaire. Potential associated factors included sociodemographic characteristics, clinical and treatment variables (e.g., hemoglobin A1c [HbA1c] and blood pressure), health behaviors, body composition metrics, and hypoglycemia-related factors. Data were analyzed using the chi-square test, the Fisher exact test, independent-sample t-tests, and stepwise regression analysis.

**Results:**

The prevalence of IAH was 13.5%. Factors significantly associated with IAH were the duration of diabetes (β = −0.38, *p* = .010) and the use of insulin degludec (β = 0.29, *p* = .015).

**Conclusion:**

These findings suggest that longer disease duration and specific treatment modalities, particularly the use of insulin degludec, are key factors associated with IAH. Accordingly, patients with long-standing T2DM should be prioritized for regular IAH screening. Furthermore, targeted nursing interventions, such as structured hypoglycemia education tailored for insulin degludec users, frequent self-monitoring of blood glucose, and individualized counseling on hypoglycemia prevention and management, are recommended to reduce the risk of IAH and promote safer glycemic control among patients with T2DM.

## INTRODUCTION

### 1. Background

Type 2 diabetes mellitus (T2DM) is a major global public health concern. According to the World Health Organization, the estimated prevalence of diabetes among adults aged ≥ 18 years was 14.0% worldwide as of 2022 [1]. Although T2DM accounts for over 90% of all diabetes cases and is largely preventable, its prevalence continues to rise [2].

Hypoglycemia, the most common acute complication of diabetes treatment, has significant short- and long-term consequences. The short-term consequences include fatal arrhythmia, brain damage, and severe physical injury [3], whereas the long-term effects involve neurocognitive impairment [4]. Hypoglycemia also exerts multifaceted effects, such as reduced cognitive function, increased cardiovascular risks, diminished quality of life, higher hospitalization rates, and elevated healthcare costs [5-9]. When hypoglycemia-induced symptoms are absent or insufficiently recognized, the condition may progress to life-threatening conditions, such as seizures, loss of consciousness, myocardial infarction, or even death [10,11]. The American Diabetes Association emphasized individualized glycemic targets and proactive hypoglycemia prevention strategies in its clinical guidelines [12].

Repeated episodes of hypoglycemia in individuals with diabetes can lead to an impaired awareness of hypoglycemia (IAH) [13]. Normally, a drop in the blood glucose level < 70 mg/dL triggers the activation of the sympathetic nervous system, producing warning symptoms, such as sweating and tremors that alert the individual to take corrective action [14]. However, in patients with IAH, this autonomic response becomes blunted, and the hypoglycemia often goes unrecognized until the blood glucose reaches dangerously low levels [15]. Consequently, IAH is associated with a markedly increased risk of severe hypoglycemia, with some studies reporting a 17-fold higher risk among affected individuals [16].

To date, most studies on IAH have primarily focused on Western populations [3–6, 16-22], individuals with Type 1 diabetes [3,4,15,18,21], or patients using insulin [4,6,14,16,17,19,20,23,24]. While these Western studies have provided valuable insights into the prevalence and predictors of IAH, their findings may not be fully generalizable to Asian populations due to significant differences in genetic background, lifestyle, and healthcare environments [16,17,25,26]. Research on IAH in Asia is relatively scarce and often restricted to insulin-treated patients, with heterogeneous measurement tools, such as the Gold score, Clarke's questionnaire, and the Pedersen-Bjergaard scale, further complicating comparability across studies. A meta-analysis of 1,726 patients with T2DM across 10 countries reported an IAH prevalence of 22.0% [27]. A systematic review identified several risk factors for IAH, including demographic factors (old age and low body mass index) and clinical factors (longer duration of diabetes, frequent hypoglycemic events, low HbA1c value due to strict glycemic control, and the use of insulin or sulfone drugs) [27]. However, these factors are not consistent across all studies and can differ by population, thus requiring additional testing for verification. Clinical guidelines based on Western results may not be appropriate, as Southeast Asians exhibit a range of etiological, educational, and cultural differences from Western populations [26]. Consequently, studies on diverse cultural populations, including Asians, and varying treatment modes are lacking [16,17,25,26], with few studies having systematically investigated the epidemiology and determinants of IAH in South Korea.

Approximately 2% of patients with T2DM receiving insulin therapy experience hypoglycemia, which is closely associated with an increased economic burden on patients [7]. In addition, analyses of the risk factors for severe hypoglycemia in T2DM have identified advanced age, insulin use, and longer disease duration as the major contributing factors [11]. However, these studies commonly focused on the occurrence of hypoglycemic episodes themselves, without systematically examining the prevalence or determinants of IAH. To address this gap, further research is needed to identify the factors associated with IAH among patients with T2DM.

### 2. Study aim

This study aimed to explore the factors associated with IAH among patients with T2DM from multiple perspectives, including sociodemographic, clinical, treatment-related, health behavior, and body composition factors. The findings are expected to contribute to developing tailored strategies to reduce hypoglycemic risk and promote optimal glycemic control.

### 3. Research question

Which sociodemographic, clinical, treatment-related, health behavior, body composition, and hypoglycemia-related factors are significantly associated with IAH among patients with T2DM?

## METHODS

### 1. Study design

This study employed a descriptive cross-sectional design to identify the factors associated with IAH in patients with T2DM.

### 2. Participants

The participants were outpatients with T2DM who visited the internal medicine department of a tertiary hospital (Jeonbuk National University Hospital) and a regional medical center (Gunsan Medical Center) in Jeonbuk Province, South Korea.

The eligibility criteria for inclusion were as follows: individuals aged ≥ 19 years, diagnosis with T2DM, and individuals undergoing treatment with oral hypoglycemic agents or insulin therapy for > 6 months. The participants were required to be able to read, write, and understand the contents of the questionnaire and to voluntarily consent to participate after being informed of the study details. The exclusion criteria were as follows: pregnant women; those with end-stage renal disease; those with a history of psychiatric disorders, such as schizophrenia or bipolar disorder; and those who had changed their medication or insulin regimen within 3 months before the survey.

The sample size was calculated using the G*Power 3.1.9.4 program (Heinrich Heine University, Düsseldorf, Germany) for multiple regression analysis with a significance level (α) of .05, a power (1-β) of .80, an effect size (f^2^) of 0.15 (medium), and 29 predictors. A minimum sample size of 184 participants was required. Considering the potential dropout rate of 10%, 205 questionnaires were distributed. After excluding 17 responses owing to incomplete or insufficient responses, 185 participants were included in the final analysis.

### 3. Instruments

Based on previous studies, factors influencing IAH include sociodemographic, clinical, and treatment characteristics; health behavior; body composition metrics; and hypoglycemia-related factors.

#### 1) Sociodemographic characteristics

The sociodemographic characteristics included age, sex, marital status, education, occupation, and income.

#### 2) Clinical and treatment characteristics

The clinical and treatment characteristics included the duration of diabetes, number of comorbidities, anemia diagnosis, duration of oral hypoglycemic agent (OHA) taken and insulin injection, as well as details on the types of OHA and insulin therapy, HbA1c levels, and blood pressure. The HbA1c levels were measured using an Afinion 2 analyzer (Abbott Laboratories, IL, USA). Blood pressure was measured twice in both arms using a manual sphygmomanometer and auscultation, and the averages of the readings were used.

#### 3) Health behaviors

The health behaviors included smoking, drinking, and physical activity.

#### 4) Body composition metrics

Body composition metrics, including body mass index (BMI), body fat percentage, body water percentage, visceral fat index, and protein mass were measured using a multi-frequency bioelectrical impedance analysis with an InBody 3.0 device (Biospace Co., Ltd., Seoul, South Korea).

#### 5) Hypoglycemia-related factors

Hypoglycemia-related factors (frequency, severity, and fear of hypoglycemia) were included in the survey. Participants reported the frequency of hypoglycemic episodes experienced within 3 months before the survey using the following options: once, twice, thrice, four times, or more than five times. The severity of hypoglycemic symptoms was assessed using the Edinburgh Hypoglycaemia Scale [28], 23-item. This tool scores the participants’ usual feelings during hypoglycemia on a 7-point Likert scale ranging from “not at all” (1) to “very much” (7). The fear of hypoglycemia was measured using the Korean version of the provided directly by the original [29] upon permission. The original tool was developed by Gonder-Frederick et al. [29]. The tool comprises 33 items, including 15 items assessing hypoglycemia avoidance behaviors and 18 items assessing hypoglycemia worries. The responses were scored on a 5-point Likert scale ranging from “never” (0) to “almost always” (4), with total scores ranging from 0 to 132. Higher scores indicate a greater fear of hypoglycemia. At the time of development, the internal consistency of the Hypoglycemia Avoidance Behavior subscale was Cronbach’s α = .85, and that of the Hypoglycemia Worry subscale was Cronbach’s α = .94. In the present study, Cronbach’s α was .81 for the Hypoglycemia Avoidance Behavior subscale and .92 for the Hypoglycemia Worry subscale.

#### 6) IAH

The Clarke score was used to assess the awareness of hypoglycemia. This instrument comprises eight items, each rated on a 0~1 Likert scale, yielding a total score of 8. Scores ≤ 3 indicate normal hypoglycemic perception n, whereas scores ≥ 4 indicate reduced hypoglycemic perception [30]. The questionnaire was translated into Korean after permission was obtained from the original developers [18]. The initial Korean draft was back-translated into English by an expert fluent in English who had not participated in the original translation process. After back-translation, researchers and experts reviewed the translated version to ensure that it accurately reflected the constructs intended in the original instrument. Subsequently, two nursing faculty members evaluated the content validity of the translated instrument using the Content Validity Index (CVI), confirming that all items had a CVI ≥ .8. Finally, a pilot test was conducted with five patients with T2DM to assess item clarity and response time, resulting in the final Korean version of the questionnaire.

### 4. Data collection

Data collection was conducted from September 18, 2023, to November 30, 2023. The researcher explained the purpose of the study to the directors of each institution in advance and obtained their cooperation and approval before proceeding. On the designated dates, the researcher and two trained research assistants provided detailed explanations of the study’s purpose, procedures, voluntary withdrawal, and confidentiality assurances. The participants signed an informed consent form before completing the self-administered questionnaire. All physical assessments, including HbA1c measurement and body composition analysis, were conducted by the two trained research assistants, who were senior nursing students. All assistants participated in on-site training sessions and practiced the study procedures under supervision to ensure consistency and minimize potential bias.

All measurements were performed at the hospital, in a pre-designated, quiet, and enclosed private room, immediately after the completion of the self-administered questionnaire. This specific environment was utilized to ensure participant privacy and minimize external disturbances that could affect the accuracy of the measurements. Specifically, HbA1c levels were determined on-site via capillary blood sampling using the Afinion 2 analyzer. For body composition assessment, participants were instructed to stand barefoot on the foot electrodes and hold the hand electrodes with their arms abducted at a 45° angle to enable multi-frequency bioelectrical impedance analysis using the InBody 3.0 device. The entire process, including the questionnaire and all physical assessments, took approximately 20–25 min. To ensure methodological rigor, all instruments used were validated, and data were collected following standardized procedures.

### 5. Data analysis

The data were analyzed using SPSS version 23.0 (IBM Corp., Armonk, NY, USA). Descriptive statistics were used to present the distribution of each variable. Differences in the IAH according to the participant characteristics were examined using univariate analyses, including the χ^2^ test, Fisher’s exact test, and independent-samples t-tests. A multiple regression analysis was conducted to identify the factors associated with IAH. Age, sex, and other significant variables in univariate analyses were included in the final regression model.

### 6. Ethical considerations

To ensure the ethical protection of the participants, this research received approval from the Institutional Review Board of Jeonbuk National University (No. JBNU 2023-07-038-001). Informed consent was obtained from all the participants prior to the survey and assessment after providing them with detailed information about the study purpose and participation procedures. The primary researcher explained all the procedures before the participants made their final decision to participate. The participants were informed that participation was voluntary and that refusal to participate would not result in any disadvantages. They were also informed of their right to withdraw at any time, that all data would be anonymized, and that the data would be destroyed after use for research and publication purposes.

## RESULTS

### 1. Characteristics of the participants

The average age of all participants was 62.54 ± 11.58 years, with a nearly equal sex distribution (49.2% men and 50.8% women). Regarding the marital status, 55.1% lived with their spouse. Overall, 64.9% of the participants had completed high school or higher education, and 48.1% of the participants were currently employed. The largest proportion of participants (43.2%) reported a monthly income of < 1 million Korean won.

In terms of the clinical and treatment-related characteristics, the majority of participants (56.8%) had been diagnosed with diabetes for > 10 years. A total of 57.3% of participants had no comorbidities, and 14.6% of participants had been diagnosed with anemia. The mean HbA1c level was 7.52 ± 1.40%, with a mean systolic blood pressure of 129.78 ± 16.18 mmHg and a mean diastolic blood pressure of 72.10 ± 11.86 mmHg. The most common duration of OHA use was ≥ 10 years (41.1%). Regarding insulin injection, 26.5% of participants had used it for < 5 years, and 47.0% had never used it. Among the OHAs, biguanides were the most commonly used (87.6%), followed by dipeptidyl peptidase 4 inhibitors (66.3%), and sulfonylureas (32.7%). Regarding medication regimens, 45.2% of participants used dual therapy, 42.3% used triple or more combinations, and 12.5% used monotherapy. Among the insulin types, insulin degludec was used by 28.6%, insulin aspart by 35.1 %, and degludec/aspart mixed formulations by 31.2% of the participants.

With respect to health behaviors, 15.7% of the participants were current smokers, 25.4% consumed alcohol, and 54.6% engaged in physical activity. The mean BMI was 25.53 ± 4.09 kg/m^2^, the mean body fat percentage was 29.53 ± 7.48%, the visceral fat index was 10.25 ± 3.90, and the mean protein mass was 9.80 ± 1.94 kg. Regarding hypoglycemia-related characteristics, 51.4% had experienced hypoglycemia 1~2 times in the past year, whereas 48.6% reported ≥ 3 episodes. The mean scores for the severity of hypoglycemia symptoms were 48.41 ± 21.33, and the mean fear of hypoglycemia score was 16.08 ± 17.28.

IAH was observed in 13.5% of the participants, with a mean IAH score of 2.14 ± 1.26. Participants with IAH had a significantly shorter duration of diabetes than those with normal perception (χ^2^ = 8.89, *p* = .025). The duration of insulin injection use also differed significantly between the groups (χ^2^ = 7.55, *p* = .048), with a higher proportion of participants in IAH having used insulin for < 5 years. Additionally, a significant group difference was observed in the use of insulin degludec (χ^2^ = 6.93, *p* = .018), with a higher proportion of insulin degludec users in the IAH group compared to the normal perception group (Table 1).

### 2. Factors influencing IAH

Age, sex, and variables that were significant in univariate analyses (duration of diabetes, period of insulin injection, and use of insulin degludec) were included in the final multiple regression model. The overall model was statistically significant (F = 4.80; *p* = .004), explaining 12.9% of the variance (adjusted R^2^ = .129). The main finding of the multivariate analysis was the significant association between a shorter duration of diabetes and a higher risk of IAH (β = –0.38, *p* = .010). This indicates that patients with a shorter history of diabetes mellitus are significantly more vulnerable to IAH. Additionally, the use of insulin degludec was also significantly associated with an increased risk of IAH (β = 0.29, *p* = .015). The detailed results of the regression model are presented in Table 2. Multicollinearity diagnostics were performed, and all tolerance values were > 0.5 and variance inflation factors were < 2, indicating no concerns regarding collinearity.

## DISCUSSION

IAH is a critical issue because it prevents patients with diabetes from recognizing hypoglycemic episodes, thereby increasing the risk of severe hypoglycemia. In this study, patients with T2DM were classified into normal and IAH groups using the Clarke score [18], and the relationships between sociodemographic, clinical, and treatment characteristics, health behavior, body composition metrics, and hypoglycemia-related factors with IAH were evaluated. Of a total of 185 participants, 13.5% (n = 25) experienced IAH. This is consistent with previous studies reporting an IAH prevalence ranging from 9.7% [19] to 34.3% [20] among patients with T2DM receiving complex insulin regimens. After adjusting for age and sex, the duration of diabetes and insulin degludec use were identified as the significant factors influencing IAH.

A shorter duration of diabetes was associated with a higher prevalence of IAH. A shorter duration of insulin therapy was associated with a higher prevalence of IAH, supporting prior findings [19,31]. Frequent hypoglycemic episodes during the early stages of diabetes management may cause the brain to adapt to hypoglycemia, reduce symptom recognition, and potentially lead to IAH even in the early stages [32]. Although the prevalence of IAH generally increases with a longer diabetes duration [19,31], factors such as intensive glycemic control [33], frequent hypoglycemic episodes [23], and differences in the approaches for diabetes management [34] can also contribute to IAH, even in patients with a shorter diabetes duration. These factors increase the risk of IAH in patients who do not receive insulin therapy [19,27]. This phenomenon is particularly pronounced during rapid HbA1c reduction [34]. In this study, the IAH group had lower HbA1c levels than the normal-awareness group, further supporting this relationship. Rapid glycemic control can lead to frequent hypoglycemic episodes, impairing the ability of the nervous system to detect hypoglycemia, and ultimately resulting in IAH [34]. These findings suggest that the prevention and management of IAH require more than simply lowering of the HbA1c levels. Frequent glucose monitoring, individualized HbA1c targets, and a cautious approach that considers the unique circumstances of each patient are essential for maintaining stable glycemic control and mitigating the risk of IAH.

The use of insulin degludec (β = 0.29, *p* = .015) was significantly associated with a higher risk of IAH in our study. Insulin degludec is a long-acting basal insulin [35]. While its pharmacological profile is widely recognized for its stable, peakless action and reduced risk of nocturnal hypoglycemia compared to older insulins [36,37], our finding suggests a potential paradoxical effect or a unique patient-related factor influencing IAH. One plausible explanation for this unexpected association relates to the clinical use and intensity of therapy. We hypothesize that the T2DM patients in our IAH group using insulin degludec might have been undergoing a relatively more aggressive or recently intensified insulin regimen to achieve strict glycemic targets, potentially resulting in more frequent, often unrecognized, mild hypoglycemia. This aligns with the concept discussed previously, where rapid or intensive glycemic control can precede IAH. Recurrent hypoglycemia, regardless of the specific insulin type, diminishes the body’s counter-regulatory hormonal response, reducing symptom recognition [21]. Furthermore, the complexity of combining insulin degludec with oral hypoglycemic agents or prandial insulins may require careful patient education and monitoring to prevent IAH [24]. Given the limited current research on the direct relationship between insulin degludec and IAH, our study provides valuable initial data that warrants cautious interpretation. Further longitudinal studies are urgently required to comprehensively understand the specific clinical and behavioral factors contributing to the increased risk of IAH in patients using this medication.

Overall, healthcare providers need to enhance patient education on IAH by providing systematic instructions aimed at improving the patients’ ability to recognize and manage hypoglycemia symptoms [22]. Regular physical activity should be emphasized as it positively affects glucose metabolism and helps reduce the risk of hypoglycemia. It is essential to periodically review pharmacotherapy, particularly for insulin users, to adjust the medication regimen and maintain optimal glycemic control while minimizing the risk of hypoglycemia [38]. As part of this management strategy, the proactive use of continuous glucose monitoring systems is crucial for monitoring real-time changes in the blood glucose levels and preemptively detecting hypoglycemic episodes [38]. Standardized tools, such as the Clarke score should be employed regularly to assess hypoglycemia awareness and to identify high-risk patients early for tailored interventions.

The findings of this study suggest crucial practical implications and directions for Basic Nursing Science, focusing on the core areas of patient safety and empowering self-care competency. Strengthening Comprehensive Assessment is paramount at the basic nursing stage. Nurses must recognize that patients undergoing intensive glycemic control or using specific insulins, such as insulin degludec, are potential risk groups for IAH, regardless of their duration of diabetes. Therefore, it is essential to utilize standardized tools from the initial stage to facilitate the early screening of IAH.

This study provides empirical evidence for basic nursing knowledge by integrating multiple factors, including *sociodemographic, clinical, treatment-related, health behavior, and body composition* of hypoglycemia care in patients with T2DM. Understanding these factors underlying IAH enables nurses to perform more accurate patient assessments, deliver individualized education, and promote safer self-management practices to prevent hypoglycemia.

While this study is limited by its cross-sectional design and relatively small sample size, future large-scale longitudinal studies are essential to identify the specific risk factors for IAH and develop effective interventions to address them. A shorter diabetes duration was unexpectedly associated with IAH and should be interpreted cautiously. The observed association between insulin degludec use and IAH also warrants careful interpretation as causal inferences cannot be drawn from the current cross-sectional design, and the argument should not rely solely on pharmacological properties. Nonetheless, this study provides valuable foundational data on the prevalence and factors influencing IAH in patients with T2DM. By including a comprehensive range of variables (e.g., sociodemographic factors, lifestyle habits, medical history, diabetes treatment-related factors, glycemic and metabolic indicators, body composition, severity of hypoglycemia symptoms, and fear of hypoglycemia scores), this study underscores its clinical significance and offers critical insights into IAH management.

## CONCLUSION

Understanding the factors associated with IAH is essential for the early identification of high-risk individuals and tailoring individualized diabetes care. This study highlights that shorter diabetes duration and the use of insulin degludec are significant contributors to IAH among patients with T2DM. Early intervention at the initial stages of diagnosis, close monitoring, and education on recognizing hypoglycemic symptoms, prevention strategies, and appropriate responses to hypoglycemic events are required. In particular, patients prescribed insulin degludec may benefit from targeted nursing interventions, including structured insulin self-management education, self-monitoring of blood glucose levels, and personalized counseling regarding the potential risk of IAH and its consequences.

## Figures and Tables

**Table 1.** Characteristics of the Participants and Differences by IAH Status (N = 185)

Characteristics	Categories	Total	Normal perception of hypoglycemia (n = 160, 86.5%)	IAH (n = 25, 13.5%)	χ^2^ or t (*p*)
Sociodemographic characteristics					
Age (years)		62.54 ± 11.58	62.56 ± 11.63	62.36 ± 11.47	0.81 (.935)
Sex	Men	91 (49.2)	79 (86.8)	12 (13.2)	0.02 (.898)
	Women	94 (50.8)	81 (86.2)	13 (13.8)	
Marital status	Alone	37 (20.0)	29 (78.4)	8 (21.6)	2.62 (.269)
	With spouse	102 (55.1)	90 (88.2)	12 (11.8)	
	With other family	46 (24.9)	41 (89.1)	5 (10.9)	
Education	< High school	65 (35.1)	56 (86.2)	9 (13.8)	0.01 (.922)
	≥ High school	120 (64.9)	104 (86.7)	16 (13.3)	
Occupation	Unemployed	96 (51.9)	83 (86.5)	13 (13.5)	0.00 (.991)
	Employed	89 (48.1)	77 (86.5)	12 (13.5)	
Income (10,000 won/month)	< 100	80 (43.2)	69 (86.2)	11 (13.8)	4.01^†^ (.247)
	≥ 100 and < 200	26 (14.1)	24 (92.3)	2 (7.7)	
	≥ 200 and < 300	34 (18.4)	26 (76.5)	8 (23.5)	
	≥ 300	45 (24.3)	41 (91.1)	4 (8.9)	
Clinical and treatment characteristics					
Duration of DM (years)	< 1	4 (2.2)	3 (75.0)	1 (25.0)	8.89^†^ (.025)
	≥ 1 and < 5	40 (21.6)	30 (75.0)	10 (25.0)	
	≥ 5 and < 10	36 (19.5)	30 (83.3)	6 (16.7)	
	≥ 10	105 (56.7)	97 (92.4)	8 (7.6)	
Number of comorbidities	None	106 (57.3)	93 (87.7)	13 (12.3)	0.76^†^ (.896)
	Do not know	9 (4.9)	8 (88.9)	1 (11.1)	
	1	48 (25.9)	40 (83.3)	8 (16.7)	
	≥ 2	22 (11.9)	19 (86.4)	3 (13.6)	
Anemia diagnosis	No	158 (85.4)	137 (86.7)	21 (13.3)	0.05^†^ (.766)
	Yes	27 (14.6)	23 (85.2)	4 (14.8)	
HbA1c level		7.52 ± 1.40	7.59 ± 1.42	7.03 ± 1.24	1.87 (.063)
Systolic blood pressure (mmHg)		129.78 ± 16.18	129.74 ± 16.16	130.08 ± 16.64	−0.10 (.922)
Diastolic blood pressure (mmHg)		72.10 ± 11.86	71.96 ± 12.15	73.00 ± 10.00	−0.41 (.685)
Duration taking OHA (years)	None	46 (24.9)	39 (84.8)	7 (15.2)	1.35 (.742)
	< 5	34 (18.4)	29 (85.3)	5 (14.7)	
	≥ 5 and < 10	29 (15.7)	24 (82.8)	5 (17.2)	
	≥ 10	76 (41.0)	68 (89.5)	8 (10.5)	
Duration of insulin injections (years)	None	87 (47.0)	72 (82.8)	15 (17.2)	7.55^†^ (.048)
	< 5	49 (26.5)	41 (83.7)	8 (16.3)	
	≥ 5 and < 10	18 (9.7)	16 (88.9)	2 (11.1)	
	≥ 10	31 (16.8)	31 (100.0)	0 (0.0)	
Types of OHA used					
Biguanides (n = 169)	No	21 (12.4)	19 (90.5)	2 (9.5)	0.53^†^ (.743)
	Yes	148 (87.6)	125 (84.5)	23 (15.5)	
SGLT2 inhibitors (n = 169)	No	125 (74.0)	108 (86.4)	17 (13.6)	0.54 (.462)
	Yes	44 (26.0)	36 (81.8)	8 (18.2)	
DPP-4 inhibitors (n = 169)	No	57 (33.7)	49 (86.0)	8 (14.0)	0.04 (.843)
	Yes	112 (66.3)	95 (84.8)	17 (15.2)	
Sulfonylureas (n = 171)	No	115 (67.3)	97 (84.3)	18 (15.7)	0.30 (.584)
	Yes	56 (32.7)	49 (87.5)	7 (12.5)	
Thiazolidinediones (n = 169)	No	159 (94.1)	135 (84.9)	24 (15.1)	0.19^†^ (1.000)
	Yes	10 (5.9)	9 (90.0)	1 (10.0)	
Alpha-glucosidase inhibitors (n = 169)	No	166 (98.2)	141 (84.9)	25 (15.1)	0.53^†^ (1.000)
	Yes	3 (1.8)	3 (100.0)	0 (0.0)	
Meglitinides (n = 169)	No	168 (99.4)	143 (85.1)	25 (14.9)	0.18^†^ (1.000)
	Yes	1 (0.6)	1 (100.0)	0 (0.0)	
Monotherapy		21 (12.5)	18 (85.7)	3 (14.3)	0.91 (.956)
Combination of 2		76 (45.2)	64 (84.2)	12 (15.8)	
Combination of 3 or more		71 (42.3)	61 (85.9)	10 (14.1)	
Types of insulin (n = 77)					
Insulin aspart	No	50 (64.9)	46 (92.0)	4 (8.0)	0.21^†^ (.691)
	Yes	27 (35.1)	24 (88.9)	3 (11.1)	
Insulin lispro	No	74 (96.1)	67 (90.5)	7 (9.5)	0.31^†^ (1.000)
	Yes	3 (3.9)	3 (100.0)	0 (0.0)	
Insulin glargine	No	75 (97.4)	68 (90.7)	7 (9.3)	0.21^†^ (1.000)
	Yes	2 (2.6)	2 (100.0)	0 (0.0)	
Insulin glargine	No	63 (81.8)	56 (88.9)	7 (11.1)	1.71^†^ (.338)
U-300	Yes	14 (18.2)	14 (100.0)	0 (0.0)	
Insulin degludec	No	55 (71.4)	53 (96.4)	2 (3.6)	6.93^†^ (.018)
	Yes	22 (28.6)	17 (77.3)	5 (22.7)	
Insulin APS 70% + Aspart 30%	No	73 (94.8)	66 (90.4)	7 (9.6)	0.42^†^ (1.000)
	Yes	4 (5.2)	4 (100.0)	0 (0.0)	
Degludec 70% + Aspart 30%	No	53 (68.8)	48 (90.6)	5 (9.4)	0.02^†^ (1.000)
	Yes	24 (31.2)	22 (91.7)	2 (8.3)	
Insulin glargine + Lixisenitide	No	75 (97.4)	68 (90.7)	7 (9.3)	0.21^†^ (1.000)
	Yes	2 (2.6)	2 (100.0)	0 (0.0)	
Insulin degludec + Lilaglutide	No	74 (96.1)	67 (90.5)	7 (9.5)	0.31^†^ (1.000)
	Yes	3 (3.9)	3 (100.0)	0 (0.0)	
Rapid and long (stand-alone)		21 (27.3)	19 (90.5)	2 (9.5)	0.76^†^ (1.000)
Mixed		20 (26.0)	18 (90.0)	2 (10.0)	
GLP-1 analogues		5 (6.5)	5 (100.0)	0 (0.0)	
Dual		22 (28.5)	20 (90.9)	2 (9.1)	
Triple or more		9 (11.7)	8 (88.9)	1 (11.1)	
Health behavior					
Smoking	No	156 (84.3)	136 (87.2)	20 (12.8)	0.39^†^ (.555)
	Yes	29 (15.7)	24 (82.8)	5 (17.2)	
Drinking	No	138 (74.6)	120 (87.0)	18 (13.0)	0.10 (.749)
	Yes	47 (25.4)	40 (85.1)	7 (14.9)	
Physical activity	No	84 (45.4)	69 (82.1)	15 (17.9)	2.48 (.115)
	Yes	101 (54.6)	91 (90.1)	10 (9.9)	
Body composition metrics					
BMI (kg/㎡)		25.53 ± 4.09	25.33 ± 4.06	26.80 ± 4.14	−0.85 (.399)
Body fat percentage (%)		29.53 ± 7.48	29.22 ± 7.34	31.56 ± 8.15	−1.46 (.146)
Body water percentage (%)		40.65 ± 10.18	40.25 ± 10.26	43.16 ± 9.45	−1.33 (.185)
Visceral fat index		10.25 ± 3.90	10.12 ± 3.91	11.12 ± 3.80	−1.20 (.233)
Protein mass (kg)		9.80 ± 1.94	9.74 ± 1.96	10.20 ± 1.81	−1.11 (.269)
Hypoglycemic-related characteristics					
Frequency of hypoglycemia (n = 109)	1~2 times	56 (51.4)	48 (85.7)	8 (14.3)	3.61^†^ (.094)
	3 times or more	53 (48.6)	51 (96.2)	2 (3.8)	
Severity of hypoglycemia symptoms		48.41 ± 21.33	48.03 ± 21.94	50.80 ± 17.12	−0.60 (.548)
Fear of hypoglycemia		16.08 ± 17.28	16.32 ± 17.23	14.56 ± 17.91	0.47 (.637)

Values are presented as the mean ± standard deviation or n (%). IAH = Impaired awareness of hypoglycemia; DM = Diabetes mellitus; HbA1c = Hemoglobin A1c; OHA = Oral hypoglycemic agent; SGLT = Sodium-glucose cotransporter; DPP = Dipeptidyl peptidase; APS = Aspart protamine suspension; GLP = Glucagon-like peptide; BMI = Body mass index.

^†^Fisher's exact test.

**Table 2.** Factors Affecting Impaired Awareness of Hypoglycemia (N = 185)

Characteristics	β	*p*	95% CI	VIF
Age	0.08	.534	−0.00~0.01	1.33
Sex^†^	0.12	.315	−0.07~0.20	1.14
Duration of DM^†^	−0.38	.010	−0.21~−0.03	1.82
Period of insulin injection^†^	0.09	.493	−0.05~0.11	1.60
Insulin degludec^†^	0.29	.015	0.04~0.33	1.15
R^2^ = .187, Adjusted R^2^ = .129, F = 4.80 (*p* = .004).				

CI = Confidence interval; VIF = Variance inflation factor; DM = Diabetes mellitus.

^†^Dummy variable: Duration of DM (ref= < 1); Sex (ref= men); Insulin degludec (ref= no); Period of insulin injection (ref= none).

## Data Availability

The data that support the findings of this study are available from the corresponding author upon reasonable request.
